# Characterisation of sleep apneas and respiratory circuitry in mice lacking CDKL5

**DOI:** 10.1111/jsr.14295

**Published:** 2024-07-24

**Authors:** Gabriele Matteoli, Sara Alvente, Stefano Bastianini, Chiara Berteotti, Elisabetta Ciani, Elenia Cinelli, Viviana Lo Martire, Giorgio Medici, Tommaso Mello, Elena Miglioranza, Alessandro Silvani, Donatella Mutolo, Giovanna Zoccoli

**Affiliations:** ^1^ Department of Biomedical and Neuromotor Sciences Alma Mater Studiorum ‐ University of Bologna Bologna Italy; ^2^ Department of Experimental and Clinical Medicine, Section of Physiology University of Florence Florence Italy; ^3^ Department of Experimental and Clinical Biochemical Sciences “Mario Serio” University of Florence Florence Italy

**Keywords:** CDKL5 deficiency disorder, mouse model, obstructive sleep apnea, preBötzinger complex, whole‐body plethysmography

## Abstract

CDKL5 deficiency disorder is a rare genetic disease caused by mutations in the *CDKL5* gene. Central apneas during wakefulness have been reported in patients with CDKL5 deficiency disorder. Studies on CDKL5‐knockout mice, a CDKL5 deficiency disorder model, reported sleep apneas, but it is still unclear whether these events are central (central sleep apnea) or obstructive (obstructive sleep apnea) and may be related to alterations of brain circuits that modulate breathing rhythm. This study aimed to discriminate central sleep apnea and obstructive sleep apnea in CDKL5‐knockout mice, and explore changes in the somatostatin neurons expressing high levels of neurokinin‐1 receptors within the preBötzinger complex. Ten adult male wild‐type and 12 CDKL5‐knockout mice underwent electrode implantation for sleep stage discrimination and diaphragmatic activity recording, and were studied using whole‐body plethysmography for 7 hr during the light (resting) period. Sleep apneas were categorised as central sleep apnea or obstructive sleep apnea based on the recorded signals. The number of somatostatin neurons in the preBötzinger complex and their neurokinin‐1 receptors expression were assessed through immunohistochemistry in a sub‐group of animals. CDKL5‐knockout mice exhibited a higher apnea occurrence rate and a greater prevalence of obstructive sleep apnea during rapid eye movement sleep, compared with wild‐type, whereas no significant difference was observed for central sleep apnea. Moreover, CDKL5‐knockout mice showed a reduced number of somatostatin neurons in the preBötzinger complex, and these neurons expressed a lower level of neurokinin‐1 receptors compared with wild‐type controls. These findings underscore the pivotal role of CDKL5 in regulating normal breathing, suggesting its potential involvement in shaping preBötzinger complex neural circuitry and controlling respiratory muscles during sleep.

## 1 INTRODUCTION

CDKL5 deficiency disorder (CDD) is a severe developmental X‐linked encephalopathy caused by mutations in the *CDKL5* gene, located on the short arm of the X chromosome (Montini et al., 1998). This gene encodes for the cyclin‐dependent kinase‐like 5 (CDKL5), a serine/threonine kinase involved in many physiological functions, especially at the brain level (Zhu & Xiong, 2019). Because of its X‐linked dominant inheritance, CDD primarily affects females, albeit with severity influenced by the random inactivation of the X chromosome, and it frequently proves fatal for males during fetal development (Jakimiec et al., 2020). Typical symptoms of CDD are drug‐refractory and early‐onset epilepsy, delayed psychomotor development, and intellectual disability (Leonard et al., 2022). Patients with CDD also have a wide range of accessory symptoms, including breathing problems such as hyperventilation and central apneas during wakefulness (Hagebeuk et al., 2013, 2023).

Brainstem dysfunction may play a role in breathing disturbances in patients with CDD (Hagebeuk et al., 2023). Breathing is generated by a complex respiratory network in the brainstem; its core is the preBötzinger complex (preBötC), an area essential for normal breathing, and widely recognised as necessary and sufficient to generate the inspiratory phase of respiration (Cinelli et al., 2021; Smith et al., 1991). PreBötC neurons also project to premotor hypoglossal neurons that modulate upper airway muscle activity during inspiration (Koizumi et al., 2008; Revill et al., 2015; Tan et al., 2010; Wang et al., 2014). A group of preBötC glutamatergic neurons express excitatory neurokinin‐1 receptors (NK1Rs), and their destruction impairs rhythmic breathing (Gray et al., 2001; McKay et al., 2005). A subset of these cells also expresses the peptide somatostatin (SST), and nearly all SST neurons in the preBötC express NK1Rs (Stornetta et al., 2003). SST neurons in the preBötC send a prominent projection to hypoglossal premotor areas (Tan et al., 2010), where they may regulate upper airway muscle activity (Chamberlin et al., 2007). SST neurons play an essential role in the generation of the breathing pattern (Ashhad & Feldman, 2020; Cui et al., 2016; Del Negro et al., 2018; Tan et al., 2008). Indeed, bilateral silencing of SST neurons in the preBötC causes persistent apnea in awake adult rats (Tan et al., 2008), whereas their photostimulation in early inspiration induces an augmented inspiratory burst, suggesting that these neurons primarily act to pattern respiratory motor output (Cui et al., 2016).

Sleep apnea is the most frequent sleep‐related breathing disorder (Maiolino et al., 2020). Sleep apnea can be classified as central (CSA), when there is an impairment in the central transmission of the signal for inspiration, or as obstructive (OSA), when an obstruction of the airway occurs (Sateia, 2014). Regrettably, information on the occurrence and characteristics of sleep‐related breathing disorders in patients with CDD remains limited due to the scarcity of investigations. A comprehensive evaluation of the sleep and respiratory phenotype was conducted in only five female paediatric patients with CDD (Hagebeuk et al., 2013, 2023). In contrast, multiple experiments were performed on mice lacking CDKL5 kinase (CDKL5‐knockout [KO]), a widely used animal model that recapitulates the core aspects of human CDD (Amendola et al., 2014), and confirmed the presence of apneas during sleep in both sexes (Fuchs et al., 2018; Gennaccaro et al., 2021; Lo Martire et al., 2017; Medici et al., 2022). It is not known, however, whether sleep apneas in CDKL5‐KO are CSA or OSA, and may be driven by alterations of brain circuits modulating breathing rhythm, such as the preBötC neurons expressing NK1Rs and SST. This study aimed to categorise, for the first time, sleep apneas into CSA and OSA in CDKL5‐KO mice by simultaneously measuring ventilation and diaphragm activity during sleep. Furthermore, immunohistochemical analyses were performed to explore, in the same CDKL5‐KO mice, possible changes in the cell number and receptor expression within the crucial preBötC neuronal population that expresses NK1Rs and SST.

## 2 METHODS

### 2.1 Ethical approval

The study protocol complied with the European Directive 2010/63/EU for animal experiments and with Italian law (authorisation n. 535/2022‐PR). Experiments were performed according to the guidelines of the Animal Welfare Committee of the University of Bologna, Italy (Legislative Decree n. 26 of 2014) and ARRIVE guidelines. All efforts were made to minimise the number of animals and their suffering.

### 2.2 Mice and experimental protocol

Experiments were performed on adult male mice carrying the mutation (hemizygous or CDKL5‐KO mice) with a C57BL/6N genetic background (*N* ≥ 3; Amendola et al., 2014) and on wild‐type (WT) male mice, used as controls. Mice were bred and housed under a 12:12‐hr light–dark cycle with ambient temperature set at 23°C, and free access to water and food in the facilities of the Department of Biomedical and Neuromotor Sciences, University of Bologna, Italy.

Surgery was performed on 12 CDKL5‐KO mice and 10 WT mice that were matched for age and weight (*p* = 0.638 and *p* = 0.197, respectively, Mann–Whitney tests). Age was 48.9 (3.1) weeks versus 50.1 (1.9) weeks, whereas weight was 28.9 (2.7) g versus 31.7 (4.3) g in CDKL5‐KO and WT mice, respectively (data expressed as median with interquartile range in brackets).

Mice were implanted with electrodes for the recording of electroencephalographic (EEG), nuchal electromyographic (nEMG) and diaphragm electromyographic (DIA) activity, as previously described (Bartolucci et al., 2021; Bastianini et al., 2014). Briefly, mice were anaesthetised with isoflurane in O_2_, and an analgesic drug (Carprofen 0.1 mg; Zoetis, Rome, Italy) and an antibiotic (benzathine benzylpenicillin and dihydrostreptomycin sulphate; MSD Animal Health, Milan, Italy) were administered. For the differential EEG signal detection, two stainless‐steel screws (2.4 mm length and 1.19 mm diameter; model 00‐96x3/32, Plastics One, Roanoke, VA, USA) were implanted into ipsilateral frontal and parietal bones through burr holes and positioned in contact with the *dura mater*. For the nEMG signal recording, two stainless‐steel wires (A‐M Systems, WA, USA) were implanted bilaterally into the neck muscles. Finally, for the detection of DIA activity, a pair of stainless‐steel wires, bent into a hairpin‐like shape and twisted to form a circular end, were inserted into the abdominal cavity and sutured in the eighth intercostal space to maintain contact with the abdominal surface of the diaphragm. The other ends of these wires, soldered to a connector (0.5 cm wide and 0.3 cm high; RS Components International, Milan, Italy), were tunnelled subcutaneously to the mouse head and fixed to the skull together with EEG and nEMG electrodes using dental cement (3M™ ESPE™ RelyX™ Unicem, 3M, Milan, Italy) and acrylic paste (DuraLay, Reliance Dental Manufacturing LLC, IL, USA).

### 2.3 Whole‐body plethysmography

After 4–5 days of individual housing for proper recovery, each mouse underwent two 7‐hr recording sessions, separated by a 24‐hr interval, inside a whole‐body plethysmography (WBP) chamber starting at lights on. Before each recording, mice were slightly anaesthetised to plug a lightweight cable for the acquisition of EEG, nEMG and DIA signals, and then inserted in the WBP chamber to simultaneously record ventilation, behavioural states and diaphragmatic activity (Bartolucci et al., 2021).

The WBP (PLY4223, Buxco, Wilmington, NC, USA) consisted of two chambers: one as a reference and one to accommodate the mouse (Bastianini et al., 2015, 2017). The animal chamber was modified by inserting a solid Plexiglas block to reduce its internal volume to 0.97 L, and was equipped with a probe to measure the chamber temperature and humidity (Rense Instruments, Rowley, MA, USA) and with a rotating electrical swivel (Plastics One, Roanoke, VA, USA) to prevent twisting of the mouse wire tether. During recordings, chambers were continuously flushed with air at 1.5 L min^−1^ to prevent CO_2_ accumulation. The respiratory signal was derived from the differential pressure between the animal and the reference chamber, measured with a high‐precision differential pressure transducer (Validyne Engineering, Northridge, CA, USA).

The EEG, nEMG and DIA signals were acquired via cable, amplified and filtered with passband filters (EEG: 0.3–100 Hz; nEMG and DIA: 100–1000 Hz) using 7P511J amplifiers (Grass, West Warwick, RI, USA). Data were digitised together with signals from the WBP using a PCI6224 board (National Instruments, Austin, TX, USA) operated by a software written in the laboratory using LabVIEW (National Instruments). The sampling frequencies were 4 Hz (WBP temperature and humidity), 256 Hz (EEG), 1024 Hz (WBP differential pressure) and 2048 Hz (nEMG and DIA). For storage, EEG, nEMG and WBP pressure signals were down‐sampled at 128 Hz, whereas the DIA signal was down‐sampled at 1024 Hz.

At the end of each recording, the system was calibrated dynamically with a 100‐μl micro‐syringe (Hamilton, Reno, NV, USA).

### 2.4 Sleep stage discrimination

The discrimination of the wake–sleep states was performed by visual analysis of raw EEG and nEMG traces on 4‐s epochs, using a validated semi‐automatic algorithm (SCOPRISM), and confirmed or corrected by trained operators, according to validated guidelines (Bastianini et al., 2014; Silvani et al., 2009). Briefly, wakefulness (W) was assigned when the nEMG tone was high and variable, and the EEG was at a low voltage with possible *θ* (6–9 Hz) and *δ* (0.5–4 Hz) frequency components; non‐rapid eye movement sleep (NREMS) was scored when the nEMG tone was lower than in W and the EEG was at a high voltage with prominent *δ* frequency components; rapid eye movement sleep (REMS) was defined in the presence of muscle atonia with occasional muscle twitches in the nEMG and of an EEG at a low voltage with predominant *θ* activity. Epochs with signals that could not be unambiguously scored were classified as “indeterminate”.

### 2.5 Analysis of respiratory variables

The analysis of respiratory variables was performed with a software developed in MATLAB (The MathWorks, Natick, MA, USA; Bastianini et al., 2015, 2017; Silvani et al., 2014). Specifically, single breaths were automatically identified from the upward (positive) WBP pressure deflection peak and errors in breath detection were manually excluded. The analysis of respiratory variables was confined to stable periods of NREMS and REMS lasting at least 12 s. The analysis of breathing and the consequent classification of apneas were not performed during W because of the movement‐related artefacts in the WBP pressure signal (Bastianini et al., 2015, 2017; Silvani et al., 2014).

Tidal volume (TV; the air volume moved during each cycle) was estimated with a specific equation (Drorbaugh & Fenn, 1955). Ventilatory period (VP; the duration of each breath) was calculated as the time interval between two successive peaks of inspiration. Minute ventilation (MV; the air volume inhaled/exhaled from lungs per minute) was calculated as the ratio between the average TV and the average VP. Then, volumes and flows were normalised expressing them per gram of body weight. Sighs (augmented breaths), which, in mice, occur almost exclusively during NREMS (Bastianini et al., 2019), were detected as breaths with a TV at least three times higher than the average TV for each mouse in NREMS. Similarly, apneas (breathing pauses) were designated as breaths with a VP at least three times longer than the average VP for each mouse in each sleep state (Bastianini et al., 2015, 2017; Silvani et al., 2014). Automatic detection of sighs and apneas was confirmed visually, based on raw respiratory tracings, by expert investigators to exclude artefacts.

### 2.6 Classification of apneas

Two different and independent apneas categorisations were both operated. First, based on the distance from a previous sigh, apneas were classified into post‐sigh (when the apneic event started within 8 s from a sigh) or spontaneous (when they started more than 8 s from the preceding sigh; Bartolucci et al., 2021; Bastianini et al., 2019). This analysis was restricted to NREMS episodes, as sighs were absent during REMS in both experimental groups, with the sole exception of one sigh recorded in one WT mouse. Second, apneas were discriminated as CSA in the case of concomitant absence of activity in WBP and DIA signals, or as OSA when a clear activation of the DIA signal was observed in the absence of WBP signal activity (Bartolucci et al., 2021). Apneas with unclear DIA signal were classified as indeterminate.

**FIGURE 1:**
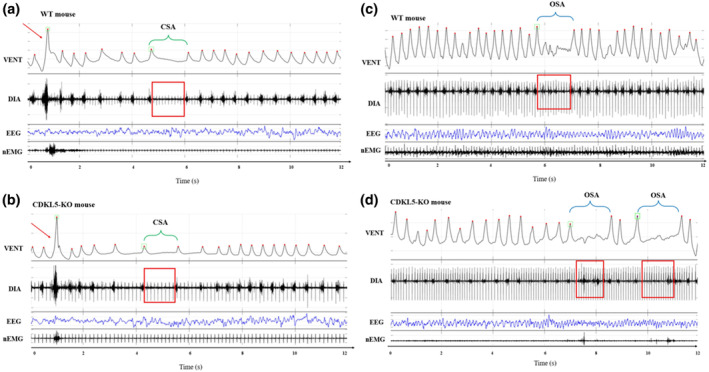
Raw tracings corresponding to central (CSA) and obstructive sleep apnea (OSA). Examples of CSA (a and b) and OSA (c and d), recorded in wild‐type (WT) and CDKL5‐knockout (CDKL5‐KO) mice within the whole‐body plethysmography (WBP) chamber. Each panel shows the differential pressure signal (VENT, representing the mouse respiratory pattern), the diaphragm electromyogram (DIA), the electroencephalogram (EEG) and the nuchal electromyogram (nEMG). In the VENT signal tracing, upward deflections indicate inspiration, red dots mark the peak of each inspiratory act, green squares indicate breaths detected as apneas or sighs, and red arrows identify sighs. Apneas are highlighted with green brackets for CSA or with blue brackets for OSA. On the DIA signal, red rectangles indicate the presence or absence of DIA burst activity.

Figure 1 shows representative examples of raw signals depicting CSA‐like and OSA‐like events in CDKL5‐KO and WT mice.

### 2.7 Analysis of heart period modulation during OSAs

The positioning of the DIA electrode also enabled recording the electrocardiographic activity superimposed on the electromyographic signal (Bartolucci et al., 2021). To explore how heart rhythm changes during OSAs, the heart period (HP) was calculated as the RR interval (i.e. the period between consecutive R wave peaks), and the difference (ΔHP) between the mean HP value during each apnea and the mean HP value over at least 10 heartbeats preceding that apnea was computed and analysed for each apnea and mouse.

### 2.8 Immunohistochemistry

At the end of the second WBP recording, mice were deeply anaesthetised with isoflurane (4%) and perfused transcardially with 50 ml of ice‐cold 0.1 m phosphate‐buffered saline (PBS; pH 7.4) followed by 200 ml of paraformaldehyde solution (4% paraformaldehyde in PBS). The brains were removed and stored overnight in the perfusion fixative at 4°C, and cryoprotected in 18% sucrose in PBS for at least 1 week. Immunohistochemical analyses were performed on six CDKL5‐KO and six WT mice.

Coronal sections (30 μm) were cut with a cryostat and stored at −20°C in an antifreeze solution for later immunohistochemical analyses. Sections were also used for histological examination of brainstem structures after staining with Cresyl violet. The atlas of Paxinos and Franklin (Paxinos & Franklin, 2007) was used to identify rostrocaudal levels and the outlines of the ventrolateral area of the medulla. Major anatomical landmarks of each investigated tissue section were compared and fitted to the anatomical structures found at the corresponding level of the atlas. The first section within a set containing the facial nucleus was considered as 0 μm. The preBötC region was located from −360 μm to −600 μm. As anatomical markers (Figure 5a,b), we used the caudal part of the hypoglossal motor nucleus, the nucleus ambiguous and the nucleus olivaris inferior. A one‐in‐two series of brain sections was used per animal, which means that each section analysed was 60 μm apart. A total of three sections comprising the preBötC (Bregma −7.0 mm) of both sides were analysed for each animal.

Free‐floating sections were rinsed (3 × 10 min) in PBS containing 0.3% Triton X‐100 (PBS‐TX), blocked with 1% bovine serum albumin (BSA; Sigma‐Aldrich, St Louis, MO, USA) in PBS‐TX for 1 hr at room temperature, and washed again three times as above. Sections were then incubated for 2 days at 4°C with a rabbit SST antibody (1:500, Cat# 20067, Lot# 2014001; ImmunoStar, Hudson, WI, USA) and a mouse anti‐NK1R antibody (1:500, Cat# 396100, Lot# UD280591; ThermoFisher Scientific, Waltham, MA, USA) dissolved in PBS‐TX containing 1% BSA. After washing in PBS‐TX (3 × 10 min), slices were subsequently incubated for 2 hr in the dark at room temperature with a mixture of Alexa Fluor 488 donkey anti‐rabbit IgG (1:200; Jackson ImmunoResearch Laboratories, Baltimore Pike, PA, USA), Alexa Fluor 568 goat anti‐mouse IgG (1:200; Invitrogen, Waltham, MA, USA) and Hoechst 33342 (10 μg ml^−1^; ThermoFisher Scientific) dissolved in PBS‐TX containing 1% BSA. After washing in PBS‐TX (3 × 10 min), tissue sections were mounted on gelatin‐coated slides and coverslipped with ProLong Gold Antifade Mountant (ThermoFisher Scientific). The specificity of antibodies against SST and NK1R has been previously reported in mice (Oliveira et al., 2019; Shi et al., 2021), and negative control staining was performed by incubating the sections in PBS‐TX containing 1% BSA without the primary antibodies.

### 2.9 Image acquisition and analysis

Images were acquired on a Leica DM6000 microscope equipped with a z‐motorised stage, a DC350FX camera (Leica, Mannheim, Germany), and the following Leica filter sets: L5 (ex 480/40 em 527/30) for Alexa Fluor 488; N2.1 (ex 515‐560, em LP590) for Alexa Fluor 568; and A4 (ex 360/40, em 470/40) for Hoechst 33342. To measure the expression of NK1Rs, all images were acquired through a 40 ×, 0.75NA HCX PL Fluotar air objective as z‐stacks in 1‐μm steps (optimised setting). Camera exposure settings were kept constant for each channel during all sessions of acquisition. SST‐positive neurons were counted within a 350‐μm circle ventral to the nucleus ambiguous, on images acquired with 10 × or 20 × objectives. Cells larger than 5 μm in diameter were counted bilaterally and the counts were averaged. Image analysis was performed using Fiji (Schindelin et al., 2012). NK1R expression was measured as integrated density in SST‐positive neurons and expressed in arbitrary units (AU). Regions of interest were manually drawn around SST‐positive neurons (Alexa Fluor 488) and the corresponding NK1R signal was measured in the Alexa Fluor 568 channel. Each SST neuron was measured once at the z‐section of maximum Alexa Fluor 488 signal intensity. Three brainstem sections containing the preBötC were analysed for each side per animal, measuring one field of view for preBötC region. Image acquisition and analysis were performed blindly with respect to the animal genotype. Images were prepared using Adobe Photoshop (Adobe, San Jose, CA, USA).

### 2.10 Statistics

Statistical analysis was performed with GraphPad Prism 9.5.1 (GraphPad Software, Boston, MA, USA). Data were first tested for normality using the Shapiro–Wilk test, controlling inflation of the type‐1 error with the false discovery rate procedure (Curran‐Everett, 2000). If the normality assumption was respected, we conducted analyses using two‐tailed unpaired *t*‐tests, one‐sample *t*‐tests, and two‐way ANOVAs with the mouse genotype (2 levels: CDKL5‐KO versus WT) and either sleep state (2 levels: NREMS or REMS) or apnea subtypes (2 levels: post‐sigh versus spontaneous) as factors, as appropriate. In case of significance of the two‐way ANOVA interaction, simple effects of the mouse genotype were assessed with independent‐sample *t*‐tests. When the normality assumption was rejected, we analysed data with Mann–Whitney tests. This was the case for the occurrence rate of either post‐sigh and spontaneous sleep apneas in NREMS, and of CSA and OSA in both NREMS and REMS. To assess the influence of OSAs on the modulation of heart rhythm in mice, ΔHP values during OSAs were analysed using a one‐sample *t*‐test with a reference value of 0. Histological data were analysed using a two‐tailed unpaired *t*‐test.

Significance was set at *p* < 0.05 and results are shown as mean ± standard error of the mean (SEM) or median (interquartile range [IQR]), as appropriate.

## 3 RESULTS

### 3.1 Analysis of sleep and respiratory variables

The analysis of sleep variables did not show any significant difference in terms of recording time spent in W, NREMS or REMS between CDKL5‐KO and WT mice (*p* = 0.506, *p* = 0.777 and *p* = 0.296, respectively, unpaired *t*‐tests; Table 1). Similarly, the analysis of VP during sleep did not reveal any significant difference (*p* = 0.219, ANOVA genotype main effect) or interaction between genotype and sleep states between CDKL5‐KO and WT mice (*p* = 0.898, ANOVA; Table 2). On the other hand, CDKL5‐KO mice had higher values of TV and MV during sleep than WT mice (*p* < 0.001 and *p* < 0.0001, respectively, ANOVA genotype main effect), with no significant interaction between genotype and sleep states (*p* = 0.147 and *p* = 0.351, for TV and MV, respectively; ANOVA; Table 2).

**TABLE 1:** Percentage of recording time spent in each state of the wake–sleep cycle in CDKL5‐KO and WT mice.

Experimental group	W (%)	NREMS (%)	REMS (%)
CDKL5‐KO (*n* = 12)	20.5 ± 1.9	68.4 ± 1.4	8.0 ± 0.5
WT (*n* = 10)	22.2 ± 1.6	68.9 ± 1.4	7.1 ± 0.7

Data were obtained during a 7‐hr recording session in the light period inside the WBP chamber. Data are expressed as mean ± SEM, and *n* represents the number of mice employed for each different experimental group.

CDKL5‐KO, CDKL5‐knockout mice; NREMS, non‐rapid eye movement sleep; REMS, rapid eye movement sleep; W, wakefulness; WT, wild‐type control mice.

**TABLE 2:** Respiratory variables during sleep in CDKL5‐KO and WT mice.

	CDKL5‐KO (*n* = 12)		WT (*n* = 10)	
Respiratory variables	NREMS	REMS	NREMS	REMS
VP (ms)	418 ± 8	375 ± 9	427 ± 7	386 ± 8
TV (μl g^−1^)^†^	9.8 ± 0.3	7.2 ± 0.2	8.2 ± 0.3	6.5 ± 0.3
MV (ml min^−1^ g^−1^)^†^	1.45 ± 0.04	1.23 ± 0.03	1.18 ± 0.06	1.06 ± 0.06
Sighs (events per hr)	14.7 ± 1.0	0.1 ± 0.1	19.0 ± 2.3	0
Post‐sigh apneas (events per hr)	1.3 (1.5)	0	0.7 (3.0)	0
Spontaneous apneas (events per hr)	1.8 (2.6)	14.2 (11.9)**	1.0 (3.1)	6.4 (6.4)

Data were obtained during a 7‐hr recording session in the light period inside the WBP chamber. Data are expressed as mean ± SEM or median (IQR) as appropriate. *n* represents the number of mice employed for each different experimental group. **p < 0.01 CDKL5‐KO versus WT (unpaired *t*‐test); ^†^ *p* < 0.001 (ANOVA genotype main effect).

CDKL5‐KO, CDKL5‐knockout mice; MV, minute ventilation (normalised to body weight); NREMS, non‐rapid eye movement sleep; REMS, rapid eye movement sleep; TV, tidal volume (normalised to body weight); VP, ventilatory period; WT, wild‐type control mice.

### 3.2 Analysis of sleep apneas

**FIGURE 2:**
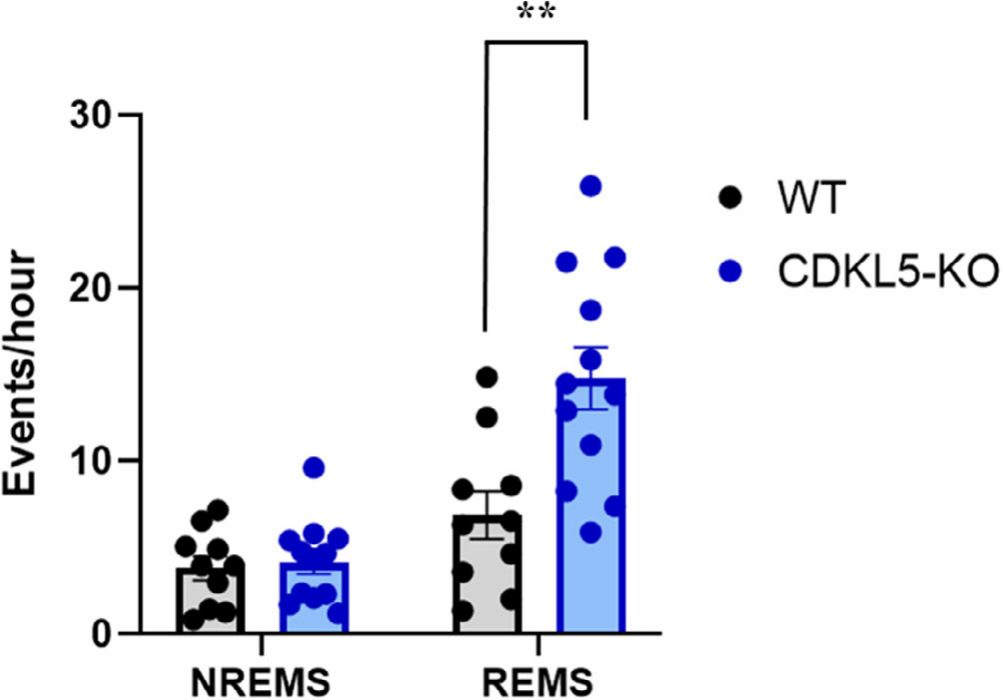
Sleep apnea occurrence rate. Data were obtained during a 7‐hr recording session in the light period inside the whole‐body plethysmography (WBP) chamber. CDKL5‐KO, CDKL5‐knockout mice; NREMS, non‐rapid eye movement sleep; REMS, rapid eye movement sleep; WT, wild‐type control mice. Data are expressed as mean ± SEM with *n* = 12 in CDKL5‐KO and *n* = 10 and WT mice. Points indicate values in individual mice. **p < 0.01 CDKL5‐KO versus WT (unpaired *t*‐test).

We found a significant interaction between genotype and sleep state for the total occurrence rate of apneas (*p* = 0.005, ANOVA). When compared with WT, CDKL5‐KO mice exhibited a significantly higher occurrence rate of apneas during REMS (6.9 ± 1.4 versus 14.8 ± 1.8 events per hr, respectively; *p* = 0.003, unpaired *t*‐test), but not during NREMS (3.8 ± 0.7 versus 4.1 ± 0.7 events per hr, respectively, *p* = 0.732, unpaired *t*‐test; Figure 2). No significant differences were found for the occurrence rate of sighs during NREMS between CDKL5‐KO and WT mice (*p* = 0.081, unpaired *t*‐test; Table 2).

The analysis of apneas during NREMS based on their temporal relationship to preceding sighs did not reveal any significant difference between CDKL5‐KO and WT mice in the occurrence rate of either post‐sigh and spontaneous apneas (*p* = 0.346 and *p* = 0.228, respectively; Mann–Whitney tests; Table 2). As expected, CDKL5‐KO mice showed a significantly higher number of spontaneous apneas during REMS with respect to WT (*p* = 0.003, unpaired *t*‐test), which was attributable to both a higher occurrence of total apneas (Figure 2) and the almost complete absence of sighs in this sleep state (Table 2).

**FIGURE 3:**
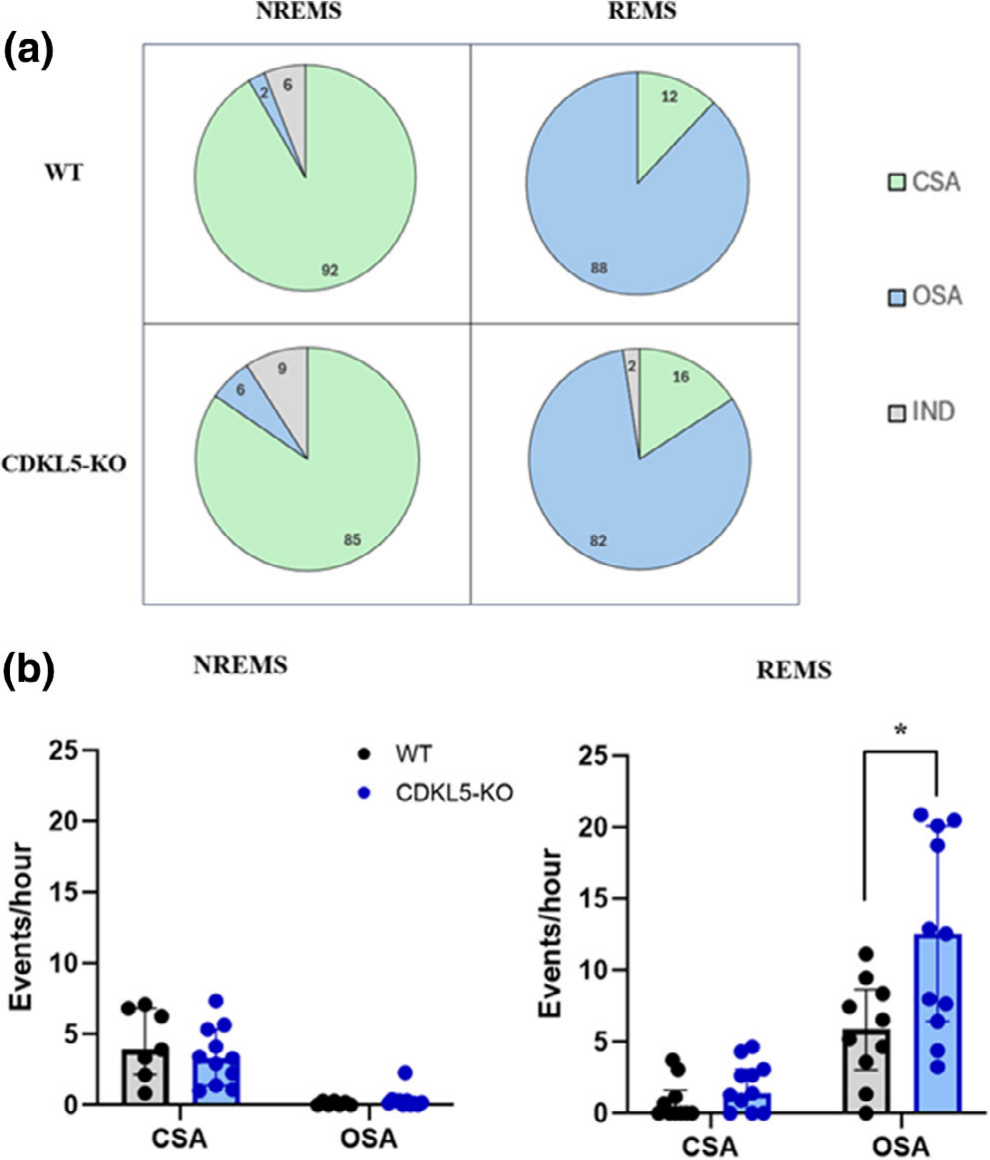
Central (CSA) and obstructive sleep apnea (OSA) occurrence rate. (a) Prevalence (expressed as percentage and indicated by numbers within the graphs) of CSA and OSA occurring during non‐rapid eye movement sleep (NREMS) or rapid eye movement sleep (REMS) in CDKL5‐knockout mice (CDKL5‐KO) and their wild‐type controls (WT) in a 7‐hr recording session during the light period inside the whole‐body plethysmography (WBP) chamber. Apneas with unclear diaphragm electromyographic (DIA) signal were classified as indeterminate (IND). (b) Occurrence rate (expressed as events per hour of sleep) of CSA and OSA occurring during NREMS and REMS. Data are expressed as median and interquartile range (IQR) with *n* = 11 in CDKL5‐KO and *n* = 7 and WT mice in NREMS, and with *n* = 11 in CDKL5‐KO and *n* = 10 and WT mice in REMS. Points indicate values in individual mice. *p < 0.05 CDKL5‐KO versus WT (Mann–Whitney test).

For the categorisation of apneic events into CSA and OSA, one CDKL5‐KO mouse was excluded from the analysis of REMS apneas, and one CDKL5‐KO mouse along with three WT mice were excluded from the analysis of NREMS apneas due to the presence of artefacts interfering with DIA evaluation. Consistent with prior research (Bartolucci et al., 2021), nearly all apneas occurring in NREMS were CSA, whereas those occurring in REMS were predominately OSA (Figure 3a).

We found no significant difference between CDKL5‐KO and WT mice during NREMS concerning the occurrence rate of either CSA or OSA (*p* = 0.536 and *p* = 0.270, respectively, Mann–Whitney tests). Specifically, we identified an occurrence rate of 3.3 (4.0) and 3.9 (4.7) events per hr for CSA in CDKL5‐KO and WT mice, respectively, and of 0.2 (0.3) and 0 (0.3) events per hr for OSA in CDKL5‐KO and WT mice, respectively (Figure 3b).

Conversely, CDKL5‐KO mice had a higher occurrence rate of OSA during REMS compared with WT controls, whereas no significant difference was observed in the occurrence rate of CSA (*p* = 0.043 and *p* = 0.116, respectively, Mann–Whitney tests). In particular, we found an occurrence rate of 12.6 (13.7) and 5.9 (5.6) events per hr for OSA in CDKL5‐KO and WT mice, respectively, and of 1.4 (3.1) and 0 (1.6) events per hr for CSA in CDKL5‐KO and WT mice, respectively (Figure 3b).

### 3.3 HP modulation during OSAs

The analysis of HP modulation during OSAs was restricted to REMS due to the rarity of OSAs detected during NREMS. It was performed on 10 CDKL5‐KO and nine WT mice, as two CDKL5‐KO and one WT mice were excluded from the analysis due to inadequate recovery of the electrocardiogram from the DIA signal.

**FIGURE 4:**
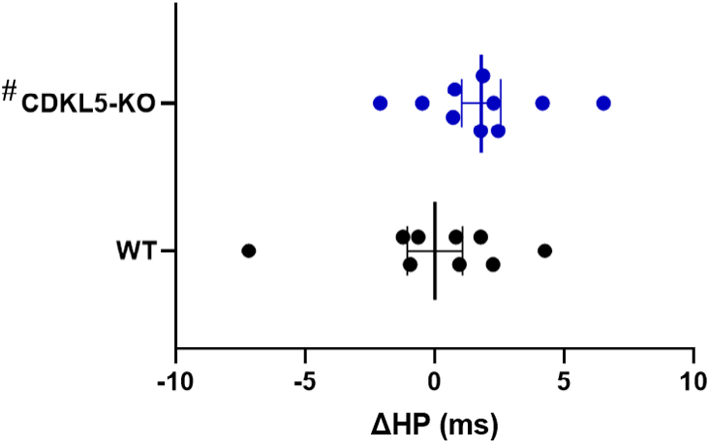
Heart period (HP) modulation during obstructive sleep apneas (OSA). Comparison of the difference (ΔHP, expressed in milliseconds) between the mean value of HP during OSA and the mean value of HP over at least 10 heartbeats preceding the apnea. All apneas were recorded during rapid eye movement sleep (REMS) in CDKL5‐knockout (CDKL5‐KO) mice and wild‐type (WT) mice in a 7‐hr recording session during the light period inside the whole‐body plethysmography (WBP) chamber. Data are expressed as mean ± SEM with *n* = 10 in CDKL5‐KO and *n* = 9 and WT mice. Points indicate values in individual mice. ^#^ *p* < 0.05 versus 0 (i.e. a significant change in HP from its baseline value before the apnea, one‐sample *t*‐test).

We found no significant differences between CDKL5‐KO and WT mice in terms of HP during OSAs (114 ± 3 versus 119 ± 4 ms, respectively) or in the immediately preceding period (113 ± 3 versus 119 ± 4 ms, respectively; *p* = 0.902, ANOVA genotype main effect). The analysis revealed a significantly positive value of ΔHP, that is, a significant increase in HP during the apnea with respect to the preceding time period in CDKL5‐KO mice, but not in WT mice (*p* = 0.042 and *p* = 0.998, respectively; one‐sample *t*‐tests versus 0; Figure 4).

### 3.4 SST neurons and their NK1R expression within the preBötC

**FIGURE 5:**
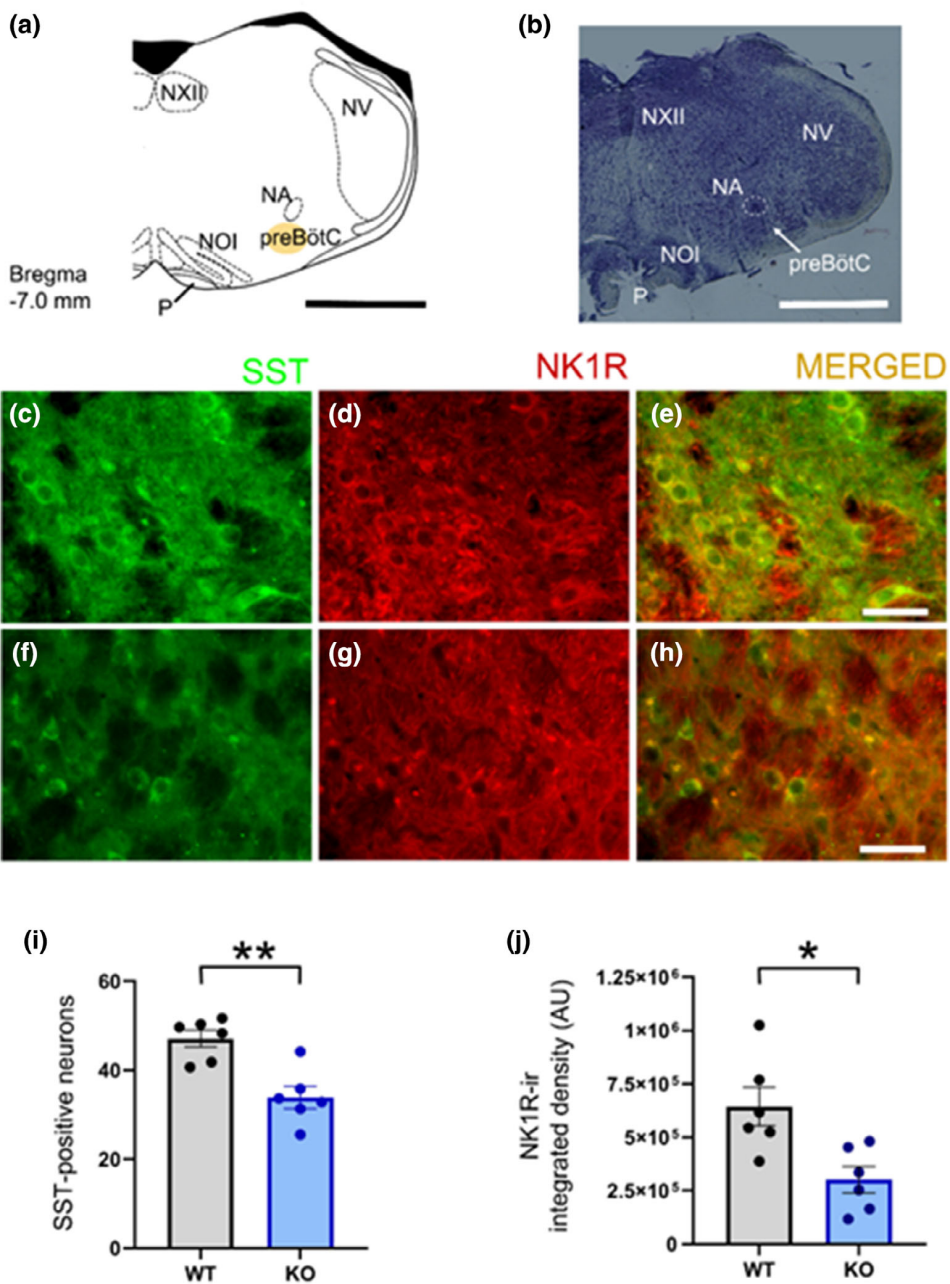
Double immunostaining and quantification of somatostatin (SST)‐positive neurons and neurokinin‐1 receptors (NK1R) immunoreactivity within the preBötzinger complex (preBötC) region. (a) Schematic representation of a coronal section of the medulla oblongata at the level of the preBötC (distance from Bregma −7.0 mm) showing the location of the analysed area (yellow circle). Outlines of the map derive from a selected section of the atlas of Paxinos and Franklin (Paxinos & Franklin, 2007) used for comparison (scale bar: 1 mm). (b) Photomicrograph of a coronal section of the medulla oblongata at the same level as in (a). The histological section is counterstained with Cresyl violet. White dashed lines surround the nucleus ambiguous (scale bar: 1 mm). NA, nucleus ambiguous; NOI, nucleus olivaris inferior; NV, nucleus tractus spinalis nervi trigemini; NXII, nucleus nervi hypoglossi; P, tractus pyramidalis; preBötC, preBötzinger complex. (c–h) Representative photomicrographs at high magnification of a coronal section of the medulla oblongata at the level of the preBötC in control (WT; c–e) and CDKL5‐knockout (CDKL5‐KO; f–h) mice showing SST‐positive neurons (green signal), NK1R expression (red signal) and merged images (scale bars: 50 μm). (i and j) Mean number of SST‐positive neurons and NK1R immunoreactivity on SST neurons expressed as integrated density (arbitrary units, AU) in the preBötC region of WT (*n* = 6) and CDKL5‐KO (*n* = 6) mice. Values are expressed as mean ± SEM, and points indicate values in individual mice. *p < 0.05; **p < 0.01 CDKL5‐KO versus WT (unpaired *t*‐test).

Representative photomicrographs showing SST‐expressing neurons (green signal), NK1R immunoreactivity (red signal), and merged images within the region corresponding to the preBötC are reported in Figure 5(c–h). We found a statistically significant decrease in the number of SST‐expressing neurons in CDKL5‐KO mice compared with WT mice (from 47.08 ± 1.91 to 33.89 ± 2.50 neurons in preBötC region, unpaired *t*‐test, *p* = 0.002; Figure 5i). Furthermore, the expression of NK1Rs in SST‐positive neurons, measured as integrated density, was significantly reduced (−53 ± 17%) in CDKL5‐KO mice (301,366 ± 60,987 AU) compared with WT (645,508 ± 91,566 AU; *p* = 0.01, unpaired *t*‐test; Figure 5j).

## 4 DISCUSSION

In the present study, we demonstrated that CDKL5‐KO mice had a significant increase in the occurrence rate of OSA during REMS compared with WT mice. In the same animals, SST neurons within the preBötC were significantly reduced in number and presented a significantly lower expression of NK1Rs with respect to WT mice.

Our research highlighted a significant increase in the occurrence rate of total apneas during REMS in CDKL5‐KO mice relative to their WT controls, with no significant differences during NREMS (Figure 2). This finding generally agrees with those of previous studies from our group on male mice carrying the mutation (male hemizygous or CDKL5‐KO mice), female mice carrying the mutation on a single X chromosome (CDKL5‐heterozygous mice), and female mice carrying the mutation on both X chromosomes (female homozygous or CDKL5‐KO mice; Fuchs et al., 2018; Gennaccaro et al., 2021;Lo Martire et al., 2017; Medici et al., 2022). In our previous studies conducted on young (14 weeks old) female CDKL5‐KO and CDKL5‐heterozygous mice and on middle‐aged (12–14 months old) male CDKL5‐KO mice, we found an increased occurrence of apneas with respect to WT mice exclusively during NREMS (Fuchs et al., 2018; Gennaccaro et al., 2021). On the other hand, our studies on young (4–6 months old) male CDKL5‐KO mice revealed an increased apnea occurrence rate during NREMS (Lo Martire et al., 2017), and either an increased or a tendency to an augmented apnea occurrence rate during REMS (Lo Martire et al., 2017; Medici et al., 2022). Taken together, these data support the view that CDKL5 deficiency increases the occurrence rate of sleep apneas. Whether such increase is restricted to NREMS or REMS or occurs in both sleep states may depend on age and sex differences of the mice, and may also reflect type‐2 statistical errors due to limited statistical power.

For the first time, in this study we categorised apneas occurring during sleep into CSA and OSA in the CDKL5‐KO mouse model of CDD. We found that CDKL5‐KO mice had an augmented occurrence rate of OSA during REMS compared with WT mice (Figure 3b). To the best of our knowledge, episodes of hyperventilation or CSA during wakefulness have been reported in patients with CDD, but there is no documented evidence of OSA in those patients (Hagebeuk et al., 2013, 2023). We are aware of a single case report of a 4‐year‐old boy with CDD experiencing sleep apnea due to hypoventilation (Mirzaa et al., 2013). Indeed, between 2003 and 2019, only about 400 individuals received a CDD diagnosis (Leonard et al., 2022) and, among them, only five young female children underwent a comprehensive and published assessment of the sleep and respiratory phenotype (Hagebeuk et al., 2013, 2023). Consequently, a connection between CDD and respiratory disturbances during sleep in humans has yet to be established.

In the present study, we also studied the HP modulation induced by OSAs. The analysis revealed a slight HP lengthening during REMS‐related OSAs in CDKL5‐KO mice, but not in WT mice (Figure 4). In humans, during OSA episodes there is a complex modulation of heart rhythm, with a reduction in heart rate (i.e. an increase in HP) both during the event (Andreas et al., 1992) and in the late inter‐apneic period (Bonsignore et al., 1997). This finding provides indirect evidence that the apneic events we detected and analysed in our mouse model of CDD are of physiological relevance. The same does not happen in WT mice, possibly due to differences in the characteristics of the OSA or in autonomic cardiac control in CDKL5‐KO mice. Accordingly, recent data uncovered cardiovascular alterations in 3–4‐month‐old CDKL5‐KO and CDKL5‐heterozygous female mice (Loi et al., 2023).

As it has been hypothesised that brainstem dysfunction may play a role in breathing disturbances in patients with CDD (Hagebeuk et al., 2023), we investigated alterations in CDKL5‐KO mice at the level of a critical area for breathing, the preBötC region. In this experiment, we focused on the excitatory glutamatergic neurons co‐expressing SST and NK1Rs within the preBötC, which are essential for the generation of breathing rhythm (Cui et al., 2016; Gray et al., 2001; McKay et al., 2005; Tan et al., 2008). A growing body of evidence from the past two decades of research has highlighted the potential relevance of SST neurons expressing NK1Rs in the preBötC in the modulation of the patency of upper airways (Chamberlin et al., 2007; Tan et al., 2010; Yang & Feldman, 2018). PreBötC SST neurons project not only to phrenic, intercostal and abdominal muscles, but also to hypoglossal premotor areas (Yang & Feldman, 2018), and the activation of NK1R‐expressing neurons in the preBötC increases rhythmic breathing and genioglossus muscle activity (Montandon et al., 2016).

Although not directly related to the present study, it should be mentioned that several lines of evidence indicate that SST functions as an inhibitory neuromodulator on breathing (see Stornetta et al., 2003; Cinelli et al., 2021 for further references). Stimulation of preBötC SST neurons results in the release of the excitatory glutamate and the co‐release of SST, which could play an inhibitory role at targets within and outside the preBötC under certain physiological or pathophysiological conditions (Yang & Feldman, 2018). The decreased number of preBötC SST neurons we found in CDKL5‐KO mice (Figure 5i) seems to be consistent with the occurrence in CDKL5‐KO mice of defects in neuronal maturation (Trazzi et al., 2016), compromised neuronal circuit connections (Wang et al., 2012) and hippocampal neurogenesis (Fuchs et al., 2014), alterations in synaptic functions of glutamatergic neurons (Zhu et al., 2023). In addition, the neuroanatomical and physiological alterations reported in the present study could be also explained by the direct interaction of the CDKL5 kinase with the methyl‐CpG binding protein 2 (MeCP2; Mari et al., 2005), whose mutation causes the typical form of Rett syndrome (Renieri et al., 2003). Indeed, this disease, partially overlapping with CDD, is a rare and well‐studied neurodevelopmental disorder encompassing the same cardiorespiratory abnormalities (apneas, breathing dysfunction and prolonged QT syndrome; Kaufmann, 2020; Tarquinio et al., 2018; Weese‐Mayer et al., 2006, 2008) recently identified in CDKL5‐KO mice (Fuchs et al., 2018; Gennaccaro et al., 2021; Lo Martire et al., 2017; Loi et al., 2023; Medici et al., 2022). Patients with Rett syndrome have a high prevalence of OSAs (Amaddeo et al., 2019; Bassett et al., 2016; Sarber et al., 2019). Further research is warranted to clarify which neural mechanisms underlie the observed respiratory anomalies during sleep in CDKL5‐KO mice.

We acknowledge our study has limitations. Experiments were performed on male mice only to decrease intra‐sample variability while limiting sample size because of the complexity of the surgical and experimental procedures. We chose to study male mice instead of female mice because the CDD course in humans is overall more severe in male than female patients, with males usually dying within their first 20 years of life (Jakimiec et al., 2020; Leonard et al., 2022). As a result of this limitation, caution is required in extrapolating our present results to female mice. Sex differences are well established in terms of dimensions of respiratory system elements (LoMauro & Aliverti, 2021), pulmonary kinematics (Torres‐Tamayo et al., 2018), ventilatory responses to hypercapnia and hypoxia (Berthon‐Jones & Sullivan, 1984; Hedemark & Kronenberg, 1982; White et al., 1982), and susceptibility to sleep‐related breathing disorders (Gargaglioni et al., 2019; Peppard et al., 2013; Young et al., 1993). Furthermore, we characterised CSA and OSA, as in our previous work on mice (Bartolucci et al., 2021), based on simultaneous recordings of WBP differential pressure and DIA. While our findings demonstrate the occurrence of alterations in breathing and respiratory muscle control during sleep in CDKL5‐KO mice, they do not clarify the correspondence of these alterations with those of human OSA. In particular, we do not know whether OSA or CSA in CDKL5‐KO mice entailed haemoglobin desaturation, and the site of transient airway obstruction in murine OSA is presently unclear. We limited our analysis of breathing to the sleep period because of movement‐related mechanical artefacts in the ventilatory signal during wakefulness (Bastianini et al., 2015, 2017; Silvani et al., 2014), whereas hyperventilation and central apneas have been reported during wakefulness in patients affected by CDD. It remains possible that sleep apneas in these patients have been underreported. Regardless, we argue that our results on mice support a potential role of CDKL5 also in controlling respiratory muscles during sleep.

Finally, as we investigated only preBötC neurons co‐expressing SST and NK1R, it remains to be investigated whether changes in other neuronal populations in the preBötC also occur in CDKL5‐KO mice. Some regions other than the preBötC could be involved in the control of upper airway muscles via hypoglossal neurons during the pre‐inspiratory phase as well as the inspiratory phase. In recent years, the pontine Kölliker‐Fuse nucleus has been shown to gate pre‐inspiratory and inspiratory motor activity of the hypoglossal nerve in adult rats (Dutschmann et al., 2021). Indeed, pre‐inspiratory discharge of the hypoglossal nerve is functionally linked to the regulation of upper airway patency as it activates both tongue protruder and retractor muscles, thereby reducing pharyngeal collapsibility and improving inspiratory airflow. The pre‐motor input of the Kölliker‐Fuse nucleus to the hypoglossal motor nucleus may underlie the Kölliker‐Fuse nucleus‐mediated gating of pre‐inspiratory/inspiratory activities of the hypoglossal nerve (Dutschmann et al., 2021). It has also been suggested that the formation of pre‐inspiratory activity in the hypoglossal nerve could directly or indirectly be affected by pre‐inspiratory neurons located in the rostral medulla including the parafacial respiratory group. In brainstem–spinal cord preparations from newborn rats, acetylcholine application induced excitatory effects on pre‐inspiratory activity in hypoglossal motoneurons consistent with changes in the burst activity of pre‐inspiratory neurons located within the parafacial respiratory group (Katsuki et al., 2021). Several neuronal groups in the lower brainstem seem to be also involved in the occurrence of CSA and OSA. Neurons of the ventral parafacial group, most often referred to as the retrotrapezoid nucleus (Guyenet & Bayliss, 2015), detect signals related to CO_2_ and/or pH levels, and generate a considerable portion of the drive to breathe during NREMS, with a possible involvement in CSA. Recent findings suggest that the nucleus tractus solitarii includes cells dually sensitive to hypercapnia and hypoxia that may be important in the detection of hypoventilation frequently seen in patients with sleep‐disordered breathing (Onimaru et al., 2021). Concerning OSA, neuromodulation of hypoglossal motoneurons during sleep is complex. Previous studies demonstrated an important role of withdrawal of excitatory serotoninergic and noradrenergic modulatory drive during REMS, arising primarily from A5 and A7 noradrenergic cell groups and serotoninergic neurons located mainly in the caudal medullary raphe (for review, see Fenik, 2015). Also, cholinergic modulation has been implicated in REMS‐related control of tongue muscle function. This cholinergic inhibitory drive could arise from the intermediate medullary reticular region (Grace et al., 2013).

## 5 CONCLUSIONS

In the present study, we found evidence of REMS‐related OSA in CDKL5‐KO mice, a model of CDD. Our data indicate that the CDKL5 kinase may play a role in the regulation of breathing, likely linked to the control of upper airway muscles. Our data also reinforce the rationale for clinical studies in the occurrence of sleep‐related breathing disorders in patients with CDD. Further research on CDKL5‐KO mice might also accelerate our understanding of the neurophysiological and molecular mechanisms that underlie REMS‐related OSA, with the potential of uncovering novel druggable targets (Alvente et al., 2023).

## AUTHOR CONTRIBUTIONS

**Gabriele Matteoli:** Investigation; formal analysis; writing – review and editing; writing – original draft. **Sara Alvente:** Investigation; writing – review and editing. **Stefano Bastianini:** Investigation; writing – review and editing; methodology; formal analysis. **Chiara Berteotti:** Investigation; writing – review and editing. **Elisabetta Ciani:** Conceptualization; methodology; writing – review and editing. **Elenia Cinelli:** Formal analysis; writing – review and editing. **Viviana Lo Martire:** Investigation; writing – review and editing. **Giorgio Medici:** Investigation; writing – review and editing. **Tommaso Mello:** Formal analysis; writing – review and editing. **Elena Miglioranza:** Investigation; writing – review and editing. **Alessandro Silvani:** Methodology; software; formal analysis; writing – review and editing. **Donatella Mutolo:** Conceptualization; methodology; supervision; writing – review and editing. **Giovanna Zoccoli:** Conceptualization; methodology; supervision; writing – review and editing; funding acquisition.

## FUNDING INFORMATION

The research was supported by the University of Bologna (RFO 2020‐2022).

## CONFLICT OF INTEREST STATEMENT

The authors declare no conflicts of interest.

## Data Availability

The data that support the findings of this study are available from the corresponding author upon reasonable request.
